# Transcription factors Elk-1 and SRF are engaged in IL1-dependent regulation of *ZC3H12A *expression

**DOI:** 10.1186/1471-2199-11-14

**Published:** 2010-02-06

**Authors:** Aneta Kasza, Paulina Wyrzykowska, Irena Horwacik, Piotr Tymoszuk, Danuta Mizgalska, Karren Palmer, Hanna Rokita, Andrew D Sharrocks, Jolanta Jura

**Affiliations:** 1 Dept of Cell Biochemistry, Jagiellonian University, Krakow, Poland; 2 Lab Of Molecular Genetics and Virology, Jagiellonian University, Krakow, Poland; 3 Faculty of Life Sciences, University of Manchester, Manchester, UK

## Abstract

**Background:**

MCPIP is a novel CCCH zinc finger protein described as an RNase engaged in the regulation of immune responses. The regulation of expression of the gene coding for MCPIP - *ZC3H12A *is poorly explored.

**Results:**

Here we report that the proinflammatory cytokine IL-1β rapidly induces the synthesis of MCPIP in primary monocyte-derived macrophages and HepG2 cells. This up-regulation takes place through the MAP kinase pathway and following activation of the transcription factor Elk-1. Using a *ZC3H12A *reporter construct we have shown that a *ZC3H12A *promoter region, stretching from -76 to +60, mediates activation by IL-1β. This region contains binding sites for Elk-1 and its partner SRF. Chromatin immunoprecipitation analysis confirms *in vivo *binding of both transcription factors to this region of the *ZC3H12A *promoter.

**Conclusions:**

We conclude that the transcription factor Elk-1 plays an important role in the activation of *ZC3H12A *expression in response to IL-1β stimulation.

## Background

MCPIP has RNase activity that prevents some immune disorders by direct control of the stability of a set of inflammatory transcripts [1,2]. MCPIP-deficient mice die within 12 weeks with the symptoms of severe inflammatory changes. Among transcripts destabilized by MCPIP are the mRNAs for IL-6, IL-12p40, calcitonin receptorand and IL-1β [1,2]. MCPIP contains a PIN domain, responsible for it's enzymatic activity and CCCH zinc finger domain, partially also engaged in the control of transcripts decay [1,2]. MCPIP is induced in human peripherial blood monocytes by monocyte chemoattractant protein (MCP-1) and this phenomenon resulted in the name of this protein as a MCP-1 inducible protein (MCPIP) [3]. The significance of MCPIP in the course of inflammation is manifested also in the development of cardiovascular diseases. Elevated level of MCPIP is associated with ischemic heart disease [3]. Recently it was found that Toll-like receptors are involved in the activation of mice *Zc3h12a *(gene encoding MCPIP). The activation of *Zc3h12a *was revealed by microarray analysis of RNA from macrophages of wild-type, Myd88^-/- ^and Trif^-/-^mice stimulated with liposacharide (LPS) [1]. We have observed that the level of transcript for MCPIP is rapidly induced by IL-1β in monocytes, fibroblasts and hepatoma HepG2 cells [2]. IL-1β is a proinflammatory cytokine produced very early in response to multiple stress. It exerts pleiotropic effects on different cell types. Binding of IL-1β to IL-1RI leads to activation of the kinase TAK1, which finally results in the activation of NFκB but also activation of MAP kinases (namely ERK, p38 and JNK) [4].

Among different transcription factors phosphorylated by MAPKs are the ternary complex factors (TCF), the subfamily of ETS-domain transcription factors [5]. The TCF subfamily include Elk-1, SAP-1 and SAP2/ERP/Net. These proteins form ternary complexes on target promoters together with MADS-box protein, serum response factor (SRF). All TCFs share the ETS-domain which is engaged in DNA-binding, the B-box responsible for association with SRF, the transcriptional-activation domain that can be phosphorylated on multiple serine and threonine residues, and the docking domain which interacts with MAPKs [5]. Elk-1 and SAP-1 are thought to act as activators whereas SAP-2 is thought to play mainly a repressive role. A series of events initiated by phosphorylation of transcription factors from the TCF subfamily are still under investigation. Phosphorylation induces conformational changes and influences the interaction of transcription factors with other co-activators/co-repressors. The details of this process are quite well understood for Elk-1. Phosphorylation leads to its de-repression through the reversing of its sumoylation and subsequent dissociation of HDAC-2 [6]. Phosphorylated Elk-1 recruits Mediator through the coactivator Sur-2/Med23 and interacts with p300/CBP which causes the enhancement of acetylation of histones of target promoters [7,8]. Recently it was also shown that histone acetylation initiated by phoshorylation of Elk-1 on the c-*fos *promoter leads to association of a second transcription factor, NFI, which in turn leads to the recruitment of the basal machinery and subsequent promoter activation [9].

The mechanisms controlling the regulation of MCPIP expression are largely unknown. Here, we have shown that IL-1β regulates expression of the gene coding for MCPIP - *ZC3H12A *through the activation of the ERK pathway. We have found that Elk-1 and SRF are transcription factors engaged in this regulation.

## Results

### IL-1β regulates the expression of ZC3H12A via activation of the NFkB and ERK pathways

**Figure 1:**
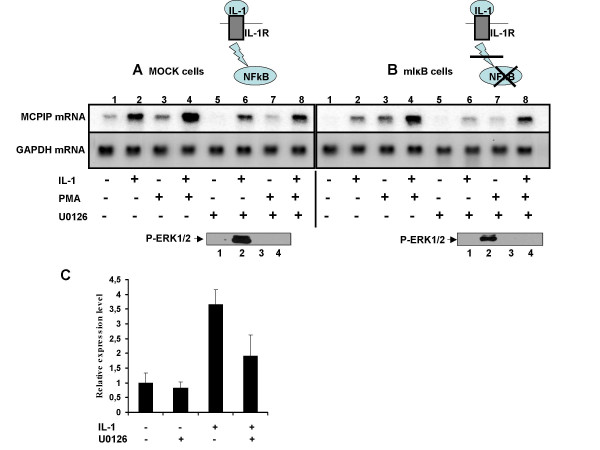
**Regulation of expression of human MCPIP**. **A**, control MOCK cells and **B**, mIκB cells with disrupted activation of NFκB were treated with IL-1β (15 ng/ml) and PMA (100 nM). To some experimental groups U0126 (10 μM) was added 30 min prior IL-1β stimulation (lanes 5-8 (A and B)). RNA was isolated after 2 h and subjected to northern blot analysis using MCPIP cDNA (top panel) or GAPDH cDNA (middle panel) as probes. Bottom panel - activation of ERK in MOCK and mIkB cells by IL-1β or its inhibition by U0126 was checked by western blot analysis. Cells were stimulated by IL-1β (15 ng/ml) for 30 min (line 2 and 4). To some experimental groups U0126 (10 μM) was added 30 min prior IL-1β stimulation (lanes 3-4). Line 1 - lysate from control cells. **C**, human macrophages were treated with IL-1β (15 ng/ml) with or without U0126 (10 μM, 30 min prior IL-1β stimulation). Changes in *ZC3H12A *expression were measured by Real Time PCR. The results are means ± SD of three independent experiments.

Our data indicate that MCPIP mRNA level is upregulated by proinflammatory cytokine IL-1β and that this regulation takes place through the activation of NFκB [10]. To test whether other pathways are engaged in the regulation of *ZC3H12A *expression we have examined the stimulation of MCPIP mRNA synthesis in a HepG2 cell line with blocked NFκB activation (mIκB cells). In this cell line NFκB is not activated by IL-1β at all whereas in both wild-type HepG2 cells and MOCK cells there is a rapid activation of NFκB (Additional file 1, Fig. S1). In mIκB cells IL-1β still increases the MCPIP mRNA level although the fold of this stimulation is much weaker than in the control (MOCK cells). Phorbol 12-myristate 13-acetate (PMA) increases the effect of IL-1β both in MOCK cells and in mIκB cells. (Fig. 1A and 1B, lane 2 and 4). These results suggest that IL-1β regulates the expression of *ZC3H12A *not only *via *activation of NFκB. In contrast to IL-1β, PMA stimulates the synthesis of MCPIP transcript in both cell lines (MOCK cells and mIκB cells) in a comparable manner (Fig. 1A and 1B, lane 3). We speculated that the possible pathway engaged in the observed stimulation of *ZC3H12A *could be the MAPK pathway. IL-1β can activate the p38, JNK and ERK kinases which phosphorylate transcription factors [11,12]. We tested the involvement of one of them, namely ERK. To examine the role of ERK in the activation of *ZC3H12A *expression we have used U0126 - a known inhibitor of MEK1/2. U0126 inhibited the activation of *ZC3H12A *by IL-1β and PMA in both MOCK and mIκB cells (Fig.1A and 1B). These observations suggest that besides NFκB activation pathway, the ERK pathway is responsible for rapid activation of *ZC3H12A *expression. ERK is activated by IL-1β in HepG2 cells and its phosphorylation is blocked when the ERK inhibitor - U0126 is present (Additional file 2, Fig. S2).

Since MCPIP plays a crucial role in the regulation of inflammation we decided to confirm the involvement of ERK pathway in the regulation of IL-1β stimulated *ZC3H12A *expression in human monocyte-derived macrophages. In macrophages treated with IL-1β the level of MCPIP mRNA was elevated and this effect was partially blocked by ERK inhibitor - U0126 (Fig. 1C). These data show that the observed mechanism of ERK-dependent regulation of *ZC3H12A *by IL-1β is not restricted to HepG2 cells.

### The ZC3H12A promoter is regulated by the transcription factor Elk-1

**Figure 2:**
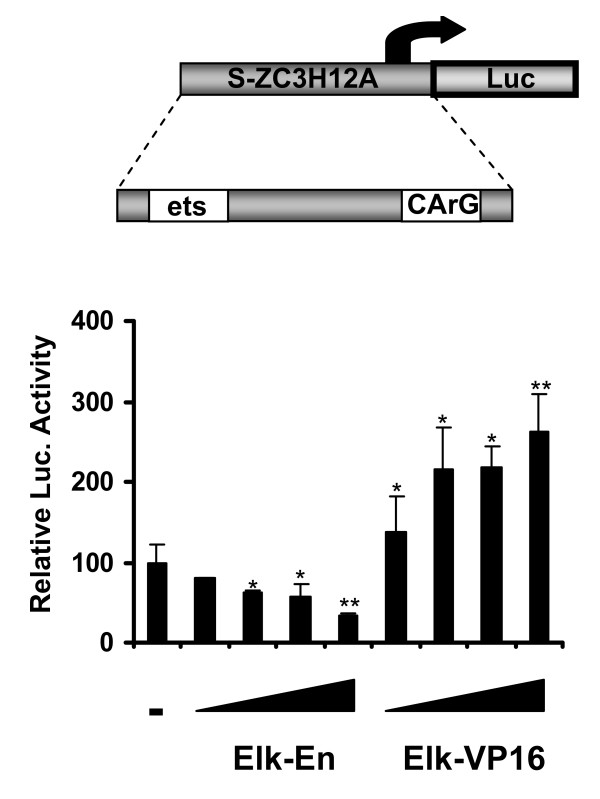
**Regulation of activation of 136 bp length *ZC3H12A *promoter fragment**. Luciferase activity was measured to examine the regulation of activation of 136 bp length *ZC3H12A *promoter fragment. HepG2 cells transiently transfected with the luciferase construct containing the fragment of human *ZC3H12A *promoter located between -76 bp and +60 bp. Increasing amounts of pElk-En (20, 50, 100, 200 ng - lanes 2, 3, 4, 5) or Elk-VP16 (20, 50, 100, 200 ng - lanes 6, 7, 8, 9) were co-transfected to the cells. Lane 1 - control cells without Elk-En or Elk-VP16. Luciferase activity was measured 24 h after transfection. Statistical significance was determined using the Student's test. *P < 0.05, **P < 0.001.

Activation of the ERK pathway leads to phosphorylation of Elk-1. To test the possible role of this transcription factor in the control of *ZC3H12A *expression we carried out transient transfection assays with increasing amounts of a repressive Elk-1 construct (Elk-En) or a constitutively active Elk-1 fusion protein (Elk-VP16) and a 2038 bp long fragment of human *ZC3H12A *promoter (-1050 - +988) - pZC3H12A-luc. Both Elk-En and Elk-VP16 regulated the activity of pZC3H12A-luc in a dose-dependent manner, with Elk-VP16 activating and Elk-En repressing as expected (Additional file 3, Fig. S3). To find sequences responsible for the observed regulation we have prepared a set of deletion mutants of the *ZC3H12A *promoter-driven luciferase reporter construct. The shortest sequence which still was responsive to Elk-1 in a dose-dependent manner turned out to be located between -76 bp and +60 bp, pS-ZC3H12A-luc (Fig. 2). Bioinformatic analysis revealed that this 136 bp long fragment contains a hypothetical ets binding site (CAGGAA) and a CArG box - SRF binding site (CCATATAAAGG). These experiments suggest that Elk-1 can contribute to the regulation of *ZC3H12A *expression. The presence of an ets binding site and a CArG box sequence suggests that the observed effect is direct.

**Figure 3:**
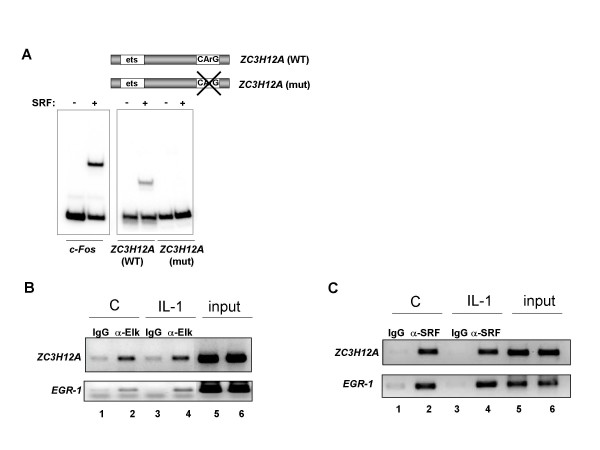
**SRF and Elk-1 bind to the *ZC3H12A *promoter**. **A**, Gel retardation assay with a fragment of *ZC3H12A *promoter (-95 - +67) containing the WT sequence or a mutated CArG box (*ZC3H12A*-mut) or a fragment of *c-Fos *promoter. The DNA was incubated with core^SRF^. **B **and **C**, Chromatin immunoprecipitation of the transcription factors Elk-1 (B) and SRF (C) bound to the *ZC3H12A *promoter. HepG2 cells were incubated in 0.5% FCS medium for 12 hrs and then stimulated for 30 min with IL-1β. The antibodies anti-Elk-1 (B) or anti-SRF (C) were used to immunoprecipitate sonicated chromatin. As a negative control non-specific IgG were employed. Eluted DNA served as a template in PCR analysis with oligonucleotides specific for *ZC3H12A *promoter (top panel B and C) or *EGR *promoter (bottom panel B and C). 1% of input DNA is shown in lines 5 and 6. The panels show the inverted images of ethidium bromide-stained gels.

The sequence of the CArG box present in the *ZC3H12A *promoter differs form the canonical one (7 instead of 6 A-T in the centre). We therefore tested whether SRF can bind to this sequence *in vitro *using a gel retardation assay. As a positive control we used a fragment of the *c-FOS *promoter (a known SRF target gene). SRF bound to the c-*FOS *promoter as expected and binding was also detected on the *ZC3H12A *promoter fragment containing a wild type CArG box, albeit to a lower level. In contrast, binding of SRF to the *ZC3H12A *promoter was not detected when the CArG box was mutated (Fig. 3A). These observations demonstrate that SRF can bind directly to the *ZC3H12A *promoter, although we were unable to detect Elk-1 binding *in vitro *(data not shown).

To demonstrate that endogenous Elk-1 and SRF can bind to the *ZC3H12A *promoter *in vivo *we carried out a chromatin immunoprecipitation experiment in HepG2 cells. In the presence of Elk-1 antibodies, the *ZC3H12A *promoter was precipitated from formaldehyde cross-linked total cell lysates (Fig. 3B, top panel, lane 2), whereas control antibodies precipitated background levels of the *ZC3H12A *promoter (Fig. 3B and 3C, top panels, lanes 1 and 3). The promoter of *ZC3H12A *was also precipitated in the presence of SRF antibodies (Fig. 3C, top panel, lane 2). IL-1β stimulation had no effect on the association of either Elk-1 or SRF with the *ZC3H12A *promoter (Fig. 3B and 3C, top panel, lane 4). Both Elk-1 and SRF were also detected as constitutively associated with the promoter of the well characterized target gene, an *EGR-1 *(Fig. 3B and 3C, bottom panels lanes 2 and 4). These observations demonstrate that endogenous Elk-1 and SRF bind to the *ZC3H12A *promoter *in vivo*, thereby demonstrating that the regulatory effects of Elk-1 and SRF on *ZC3H12A *expression are likely direct.

### Stimulation of HepG2 cells by IL-1β leads to phosphorylation of Elk-1

**Figure 4:**
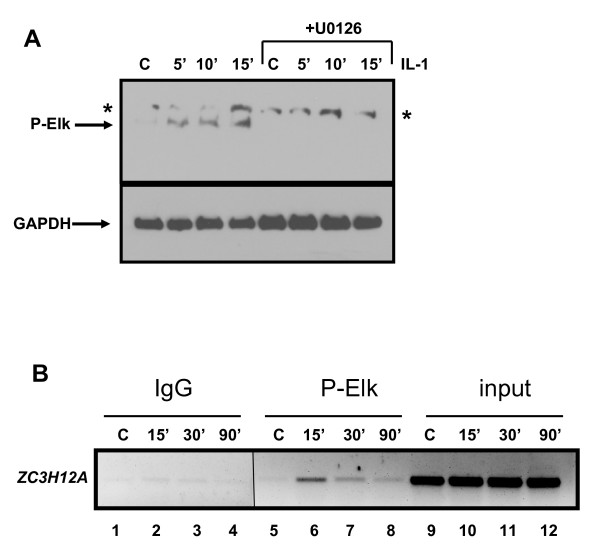
**IL-1β causes ERK-dependent phosphorylation of Elk-1 on *ZC3H12A *promoter**. **A**, Activation of Elk-1 after IL-1β stimulation. HepG2 cells were stimulated by IL-1β (15 ng/ml) for 5, 10 and 15 min and the phosphorylation of Elk-1 was analyzed by western blot analysis. To some experimental groups U0126 (10 μM) was added 30 min prior IL-1β stimulation (lanes 5-8). Asterisks indicate unspecific bands. **B**, Chromatin immunoprecipitation of the phosphorylated Elk-1 bound to the *ZC3H12A *promoter. HepG2 cells were incubated in 0.5% FCS medium for 12 hrs and then stimulated for 15, 30 or 90 min with IL-1β. The antibodies anti-P-Elk were used to immunoprecipitate sonicated chromatin. As a negative control non-specific IgG were employed. Eluted DNA served as a template in PCR analysis with oligonucleotides specific for *ZC3H12A *promoter. 1% of input DNA is shown in lines 9-12. The panels show the inverted images of ethidium bromide-stained gels.

Phosphorylation of Elk-1 is crucial for its activity. To confirm that stimulation by IL-1β induces the phosphorylation of Elk-1 through MAPK pathway we performed western blot analysis using an antibody against phosphorylated Elk-1. IL-1β induced phosphorylation of Elk-1 after 5-15 min of stimulation and this modification was blocked when the ERK pathway inhibitor - U0126 was present (Fig. 4A). Therefore in HepG2 cells stimulation by IL-1β causes phosphorylation of Elk-1 through activation of ERK.

Elk-1 is bound to the promoters of genes independently of its activation by the MAPK pathway [13,9]. The crucial factor which changes the state of the Elk-1 on the promoters and induces events leading to activation of the genes regulated by this transcription factor is phosphorylation carried out by ERK, JNK or p38. To test whether active, phosphorylated forms of Elk-1 could be detected on the *ZC3H12A *promoter after IL-1β stimulation, we performed chromatin immunoprecipitation using anti-phospho-Elk-1 (P-Ser383) antibody. Phosphorylated Elk-1 could be detected on the *ZC3H12A *promoter after 15 min treatment with IL-1β (Fig. 4B).

Taken together, these results demonstrate that IL-1β treatment leads to the increase of Elk-1 phosphorylation in an ERK pathway-dependent manner and the active phosphorylated form can be found associated with the *ZC3H12A *promoter.

### The ZC3H12A promoter is regulated by IL-1β via the ERK MAPK pathway

**Figure 5:**
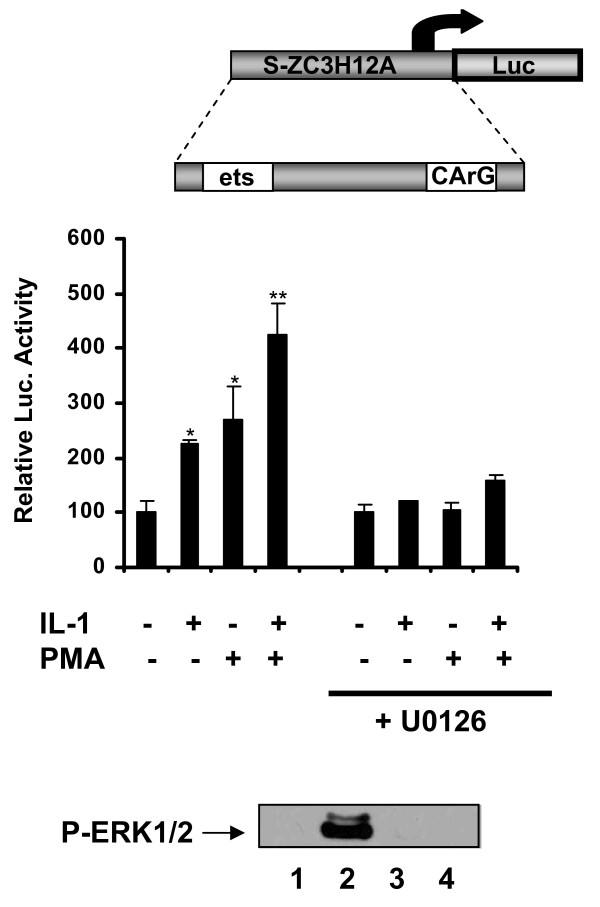
**Regulation of activation of 136 bp length *ZC3H12A *promoter fragment**. Luciferase activity was measured to examine the regulation of activation of 136 bp length *ZC3H12A *promoter fragment. HepG2 cells were transiently transfected with the luciferase construct containing the fragment of human *ZC3H12A *promoter located between -76 bp and +60 bp. 24 hrs after transfection cells were stimulated with IL-1β or/and PMA for 3 hrs. An inhibitor U0126 was added to cells 30 min prior stimulation. Statistical significance was determined using the Student's test. *P < 0.05, **P < 0.001. Bottom panel - activation of ERK by IL-1β or its inhibition by U0126 was checked by western blot analysis Cells were stimulated by IL-1β (15 ng/ml) for 30 min (line 2 and 4). To some experimental groups U0126 (10 μM) was added 30 min prior IL-1β stimulation (lanes 3-4). Line 1 - lysate from control cells.

To verify the importance of the cloned 136 bp long promoter in the regulation of *ZC3H12A *expression by IL-1β we have examined its activation by this proinflammatory cytokine. The 136 bp long promoter was activated by IL-1β and this activation was blocked by the ERK pathway inhibitor - U0126 (Fig. 5). Also PMA activated this promoter and the combination of both factors had an even greater effect (Fig. 5). These data are broadly in agreement with the data obtained by northern blot analysis (Fig. 1A and 1B). In all cases the ERK inhibitor strongly reduced the observed activation. These results confirm the involvement of the ERK pathway in the regulation of *ZC3H12A *expression by IL-1β and demonstrate the importance of the 136 bp long promoter sequence in this regulation.

### Functional analysis of ets binding site and CArG box in the ZC3H12A promoter

**Figure 6:**
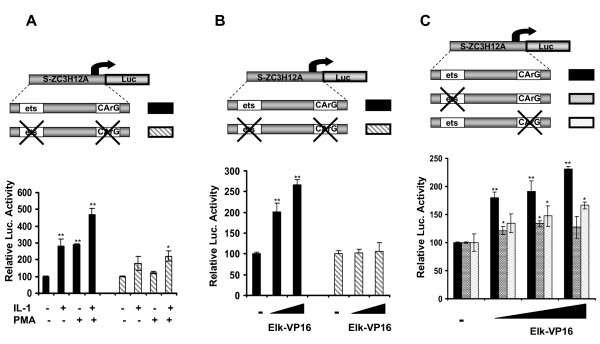
**Mutation of both ets-binding site and CArG box abolished the activation of 136 bp length *ZC3H12A *promoter fragment**. Luciferase activity was measured to examine the regulation of 136 bp length *ZC3H12A *promoter fragment. HepG2 cells were transiently transfected with the luciferase construct containing the fragment of human *ZC3H12A *promoter located between -76 bp and +60 bp. Black bars - wild type promoter, striped bars - promoter with the mutation in both ets-binding site and in CArG box. **A, **24 h after transfection cells were stimulated with IL-1β and PMA for 3 hrs. **B, **Cells were co-transfected with increasing amounts of Elk-VP16 (20, 50 ng). Luciferase activity was measured 24 h after transfection. **C, **Influence of mutation of ets-binding site or CArG box on the activation of 136 bp by Elk-1. Black bars represent wild type promoter, dotted bars represent promoter with the mutation in ets-binding site, light bars represent promoter with the mutation in CArG box. Cells were co-transfected with increasing amounts of Elk-VP16 (10, 20, 50 ng). Luciferase activity was measured 24 h after transfection. Statistical significance was determined using the Student's test. *P < 0.05, **P < 0.001.

The sequence from the human *ZC3H12A *promoter located between -76 bp and +60 bp contains an ets binding site and CArG box, sequences which hypothetically can bind Elk-1 and its partner SRF. To evaluate the contribution of these sequences to the observed regulation by Elk-1 and SRF we introduced point mutations that abolished binding of Elk-1 or SRF to these elements. We first assessed the response of this mutant promoter to activation by IL-1β. The responsiveness of the mutant *ZC3H12A *promoter to IL-1β was strongly reduced in comparison to a reporter construct containing the wild-type promoter sequence (Fig. 6A). However, the activation of the 136 bp promoter sequence, without a functional ets binding site and functional CArG box, by IL-1β was not completely blocked because this fragment still contains two NFκB binding sites [Skalniak unpublished]. This data confirm the importance of the ets binding site and the CArG box in the regulation of *ZC3H12A *expression by IL-1β.

To confirm that the mutant promoter was unresponsive to Elk-1, we examined its activation by the potent Elk-VP16 fusion protein. In comparison to the wild-type promoter, the reporter construct containing the mutated ets binding site and the mutated CArG box was not responsive to Elk-VP16 (Fig. 6B). Mutation of either the Elk-1 or the SRF binding sites was sufficient to abolish activation of the promoter by Elk-VP16 (Fig. 6C). This is consistent with a requirement for SRF to recruit Elk-1.

Together these data therefore demonstrate the importance of the ets and SRF binding elements in promoter responsiveness to Elk-1 activity and activation by IL-1β.

## Discussion

Elk-1 is a known regulator of the expression of immediate-early genes, mainly transcription factors, which in turn regulate the expression of other genes coding for proteins engaged in the response of the cell to the changing environment. Thus, activation of Elk-1 is at the top of events leading to a global changes in the cell behavior. Here we have identified a new Elk-1 target gene, *ZC3H12A*, that encodes a recently discovered protein whose biological function is the control of mRNA turn-over. Transcripts for IL-6, IL-12p40 and the calcitonin receptor are found to be regulated by MCPIP [1] and we have recently found that MCPIP regulates the turn-over of IL-1β mRNA [2]. The latter finding is intriguing and suggests the existence of an autoregulatory loop as here we show the importance of IL-1β in controlling the expression of *ZC3H12A*. The results indicate the existence of a negative regulatory loop, contributing to shut down of IL-1β synthesis.

The mechanisms controlling the regulation of the expression of *ZC3H12A *are not known. *ZC3H12A *is an immediate-early gene regulated by the proinflammatory cytokine IL-1β [2] through the activation of NFκB (Fig. 1; [10]). However in the mIκB cells with a defect in the activation of NFκB, IL-1β is still able to elevate MCPIP mRNA level (Fig. 1B). Our results suggest that apart from the NFκB activation pathway, the MAPK pathway is also engaged in the regulation of *ZC3H12A *expression. *ZC3H12A *promoter deletion constructs allowed us to find a minimal promoter 136 bp fragment which is still responsive to MAPK pathway activation. Bioinformatic analysis revealed that this region contains a hypothetical Elk-1 binding site (ets-binding site) - CAGGAA. The sequences recognized by Elk-1 differ amongst binding sites. The core GGA is always included in the ets-binding site but the flanking residues may differ. The most frequent motif is CCGGAA (in promoters of the Elk-1-regulated genes such as *EGR-1, TR3, Pip92, MCL-1, SRF*) [14,15,13]. However, other known Elk-1 target genes like c-*FOS *possess the sequence CAGGAT, whereas another one *nur77*, contains the GAGGAA motif [16,14]. Elk-1 as a member of the TCF subfamily can form ternary complexes on target promoters together with serum response factor (SRF). Indeed, there is a CArG box, an SRF binding site, in the 136 bp fragment of *ZC3H12A *promoter (CCATATAAAGG). The consensus CArG box sequence is CC(A/T)_6_GG although there are exceptions from this rule. For example *TR3 *has CCTGTATGG and *nur77 *CTATTTATAG [14]. The lack of one of the G:C base pairs flanking the central hexameric A/T rich region probably explains the lower level of binding we observe in comparison to the *c-FOS *promoter, which contains a canonical binding motif. However, the relative *in vitro *binding affinity and match to the consensus does not necessarily correlate with *in vivo *occupancy level as demonstrated by ChIP-chip analysis [17]. Indeed, we detect SRF binding by ChIP analysis to this promoter. Simultaneous mutation of both, the ets-binding site and CArG box completely blocked activation of 136 bp promoter fragment by either a constitutively active form of Elk-1 (Elk-VP16) or by PMA treatment. Activation of this fragment by IL-1β is strongly reduced although not blocked completely and this remaining responsiveness to IL-1β is probably due to two hypothetical NFkB binding sites still present in the 136 bp promoter fragment [Skalniak unpublished]. All these results suggest involvement of the ERK MAPK pathway leading to activation of Elk-1 in the regulation of *ZC3H12A *expression by IL-1β. Indeed, we demonstrate binding of Elk-1 to *ZC3H12A *promoter *in vivo *through ChIP analysis. Elk-1 binding to the *ZC3H12A *promoter is detectable in the presence and absence of stimulation with IL-1β, thus, changes in promoter occupancy does not appear to be the activation mechanism. Binding of Elk-1 to ets-binding sites of other genes in unstimulated cells was reported earlier [13]. Such binding is not sufficient for activation of genes regulated by Elk-1 since Elk-1 in unstimulated cells is sumoylated and interacts with HDAC-2. This modification keeps Elk-1 in a repressive form [6]. Our data show that IL-1β induces phosphorylation of Elk-1 and phosphorylated Elk-1 is present on the *ZC3H12A *promoter (Fig. 4). The Elk-1 partner protein, SRF, is also bound to the *ZC3H12A *promoter and this binding is also not increased upon IL-1β stimulation indicating again that transcription factor recruitment does not appear to be a key regulatory event (Fig. 3C). This seems to be a more general mechanism since the occupancy of *EGR-1 *promoter by SRF and Elk-1 is also independent of IL-1β stimulation. It has to be noticed that apart from the NFκB activation pathway and the ERK pathway another yet unknown pathway contributes to the regulation of *ZC3H12A *expression. In mIκB cells treated with the ERK inhibitor the activation of *ZC3H12A *expression by IL-1β is still observed (Fig. 1). Our preliminary data indicate that p38 and JNK could participate in this process.

## Conclusions

In summary, our results demonstrate a role of Elk-1 in the regulation of the expression of MCPIP - an RNAse important in inflammation. Until now, Elk-1 was generally thought to be involved in regulation of proliferation and apoptosis [18]. Our discovery has therefore potentially broadened the role of Elk-1 as factor which also controls the course of inflammation.

**Figure 7:**
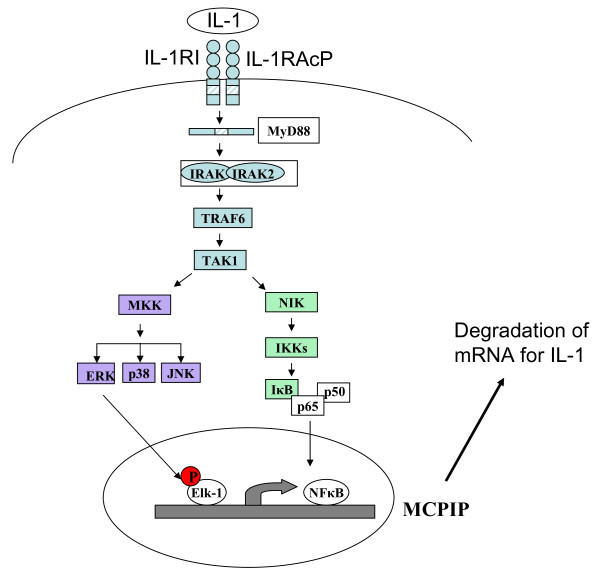
**Schematic diagram of regulation of the *ZC3H12A *promoter by IL-1β**. IL-1RI - type I IL-1 receptor, IL-1R AcP - IL-1 receptor accessory protein. The main sequences responsible for binding of NFκB are located within the second intron of *ZC3H12A *promoter [10].

Our results reveal also existence of negative regulatory loop controlling the synthesis of IL-1β. IL-1β regulates the expression of MCPIP, an RNase which contributes to the turn-over of IL-1β mRNA (Fig. 7).

## Methods

### Cell culture

HepG2 cells (ATCC), MOCK cells and mIκB cells were cultured at 37°C and 5%CO_2 _in Dulbecco Modified Eagle Medium (DMEM) with 1000 mg/l D-glucose (Gibco/BRL) supplemented with 10% foetal bovine serum (FBS). HepG2 cells stably transfected with retroviral vector pCFG5-IEG2, containing a nondegradable mutant form of IκBα (mIκB cells), and cells with an empty vector (control, MOCK cells) were used for determination of significance of NFκB signalling pathway in IL-1-dependednt activation of *ZC3H12A*. The transfected cells were kindly provided by Professor Stephan Ludwig (Heinrich-Heine University, Duesseldorf, Germany [19].

Human monocyte-derived macrophages (hMDMs) were separated from fractions of peripheral blood mononuclear cells (PBMCs) obtained from the blood of healthy donors using a lymphocyte separation medium (LSM; PAA) density gradient [20]. Briefly, isolated PBMCs were seeded 2 × 10^7 ^/well in 6-well plates (Sarstedt) in RPMI1640 (PAA) containing 2 mM L-glutamine, 50 μg ml^-1 ^gentamycin (Sigma), and 10% heat-inactivated autologous human plasma. After 24 h non-adherent cells were removed and remaining adherent monocytes were cultured for 7 days with fresh medium added every second day. The amount of serum was diminished to 0,5% 16 h before stimulation. hMDMs were stimulated with IL-1β (15ng/ml) for 2 h. When necessary an inhibitor U0126 was added 0,5 h before the cytokine stimulation.

### Cytokine and cell stimulation

Cells were stimulated with 15 ng/ml IL-1β (R&D), 100 nM PMA (Calbiochem). When applied, the inhibitor of MEK1/2, U0126 (10 μM) (Calbiochem) was added to the medium at 30 min prior stimulation.

### RNA preparation, Northern blot analysis and Real Time PCR

Total RNA was prepared using Chomczynski method with modifications as described before [21]. Ten microgram samples of RNA were subjected to formaldehyde gel electrophoresis and northern blot analysis was carried out as described previously [18]. For RT-PCR the first strand of cDNA was synthesized from 2 μg of total RNA using MMLV reverse transcriptase (Promega) and oligo(dT) primer. Real time PCR was performed using the SYBR Green PCR Master Mix (DyNAmo™ HS SYBR Green qPCR (Finnzyme). Each sample was normalized to reference gene (elongation factor 2) and the relative level of transcripts was quantified by ΔΔC_T _method.

### Nuclear extract preparation and EMSA test

HepG2, MOCK cells and mIκB cells were stimulated with IL-1β for 90 min Nuclear extracts from stimulated and unstimulated cells were prepared as described previously [22]. For NFκB binding assay double-stranded probes were labeled by filling in 5' protruding ends with Klenow enzyme using [α-^32^P]dCTP (3000 Ci/mmol). After purification with Qiagen system, the probes (10000-20000 cpm of ^32^P-labelled NFκB-binding oligonucleotide: 5'agcttcagaggggactttccgagagg) were incubated with 10 μg of nuclear extracts for 30 min at room temperature. For SRF binding gel retardation assay was carried out with ^32^P-labeled 162-base pair fragment of human *ZC3H12A *promoter generated by PCR on templates pS-ZC3H12A-luc (WT) and pS-ZC3H12A-luc (mCArG) (primers: GCCGCGACGCGAGGAGCGG and GTCCTGGGGGTAAGGACGGCG) as described previously [13]. Core^SRF ^was produced as a glutathione S-transferase-tagged protein in bacteria.

### Plasmid constructs

pEF1/Myc-His/lacZ is a control vector containing the gene for β-galactosidase (Invitrogen). pElk-VP16 is a Rous sarcoma virus promoter-driven vector encoding full-length wild type Elk-1 fused to residue 410-490 of the VP16 C-terminal sequence [22,13]. pElk-En is a CMV promoter-driven vector encoding full-length wild type Elk-1 fused to residue 2-298 of the engrailed repressor domain [13]. pZC3H12A-luc, containing 2038 bp long fragment of human *ZC3H12A *promoter (-1050 - +988), was generated by PCR cloning of this fragment to the pGL4 reporter vector (Promega). The 2038 bp fragment of human *ZC3H12A *promoter was obtained by two step PCR, using total DNA isolated from HepG2 cells. The first round PCR was carried out with the primer forward: GTGGCTCTGTCCTCCAGCGTGT and reverse: CTGGCTTCCAGGACAGGCTTC. Then the second round of PCR was performed with nested primers: forward with restriction site for XhoI: CCGCTCGAGCTCCAGCGTGTGGGCTCTGTG and reverse with restriction site for HindIII and mutated initiation of translation codon ATG: CCCAAGCTTGCCACTGATAGCTCAGACTCCTG The introduced restriction sites were used during cloning of PCR product into the pGL4 vector. pS-ZC3H12A-luc, containing 136 bp long fragment of human *ZC3H12A *promoter (-76 - + 60) was generated using PCR product obtained with the following primers: forward with XhoI restriction site: CTCGAGAGCAGGAAGGGGCGAGGCAGCC and reverse with HindIII restriction site: GGGAAGCTTCGGCGGCGCCTTTATATGGGGCG and using pZC3H12A-luc as a template. All genetic constructs were sequenced before transfection experiments. The constructs containing mutations in ets-binding site or CArG box were generated using QuickChange Site-Directed Mutagenesis kit (Stratagene) according to the manufacturer's procedure. The sequence GCAGGAA was changed to GaAttcA, the sequence CCATATAAAGGC was changed to CCATATgaattc, both mutations generated EcoRI restriction site.

### Reporter gene assay

Transient transfection experiments were carried out using Lipofectamine 2000 reagent (Invitrogen) in 12-weel plate. Total amount of 1.6 μg of DNA per each well was used, including 0.4 μg of pZC3H12A-luc or pS-ZC3H12A-luc and 10 ng of pEF1/Myc-His/lacZ. For some experiments indicated amounts of pElk-En or Elk-VP16 were used. The amount of DNA per well was equalized using mock DNA (pcDNA3). Luciferase assays were carried out using the dual light reporter gene assay system (Tropix) according to the manufacturer's procedure. β-galactosidase activity was measured to normalized the efficiency of transfection. All experiments were repeated at least three times in duplicates.

### Western blot

Western blot was carried out using Immobilon Western chemiluminescent HRP substrate (Millipore) and antibodies anti-phospho-Elk-1, anti-phospho-ERK (Santa Cruz) and anti-GAPDH (Abcam).

### Chromatin immunoprecipitation

Chromatin immunoprecipitations were carried out as described previously [16], using anti-Elk-1, anti-P-Elk (P-Ser383) and anti-SRF (Santa Cruz) or nonspecific IgG (Upstate) antibodies. Promoter-specific primers were used to amplify the DNA by PCR: human *ZC3H12A *promoter: forward: CAGGTGCGTGTACCTGATTC and rewerse: CGAGTCCTGGGGGTAAGG, and human *egr-1 *promoter: forward: TGCAGGATGGAGGTGCC and reverse: AGTTCTGCGCGCTGGGATCTC. Where indicated cells were stimulated 30 min by IL-1β (15 ng/ml).

## Authors' contributions

AK experimental design, constructs generation, reporter gene assay, chromatin immunoprecipitation, interpretation of study, writing of manuscript. PW northern blots and western blots. IH chromatin immunoprecipitation. PT western blots. DM isolation of hMDMs and RT-PCR. KP EMSA. HR, ADS and JJ interpretation of study, discussion of experimental results, manuscript revision. All authors drafted, read and approved the manuscript.

## Supplementary Material

Additional file 1 **Fig. S1. Activation of NFκB in HepG2, MOCK and mIκB cells**. Nuclear extract was isolated from HepG2 cells, MOCK cells and mIκB cells. Where indicated cells were stimulated with IL-1β (15 ng/ml). By gel retardation assay NFκB activation was measured. Click here for file

Additional file 2 **Fig. S2. Activation of ERK by IL-1β in HepG2 cells**. HepG2 cells were stimulated by IL-1β (15 ng/ml) for 15 and 30 min and the phosphorylation of ERK1/2 was analyzed by western blot analysis. To some experimental groups U0126 (10 μM) was added 30 min prior IL-1β stimulation (lanes 4-6) Click here for file.

Additional file 3 **Fig. S3. Regulation of 2038 bp length *ZC3H12A *promoter fragment by Elk-1**. HepG2 cells were transiently transfected with the luciferase construct containing fragment of human *ZC3H12A *promoter located between -1050 and +988 and increasing amounts of pElk-En (20, 50, 100, 200 ng - lanes 2, 3, 4, 5) or Elk-VP16 (20, 50, 100, 200 ng - lanes 6, 7, 8, 9). Lane 1 - control cells without Elk-En or Elk-VP16. Luciferase activity was measured 24 h after transfection. Statistical significance was determined using the Student's test. *P < 0.05, **P < 0.001. Click here for file

## References

[B1] Matsushita K Takeuchi O Standley DM Kumagai Y Kawagoe T Miyake T Satoh T Kato H Tsujimura T Nakamura H Akira S ZC3H12A is an RNase essential for controlling immune responses by regulating mRNA decay Nature 2009 458 1185 90 10.1038/nature07924 19322177

[B2] Mizgalska D Wκgrzyn P Murzyn K Kasza A Koj A Jura J Jarząb J Jura J Interleukin-1-inducible MCPIP protein has structural and functional properties of RNase participating in degradation of IL-1-mRNA FEBS J 2009 276 7386 7399 10.1111/j.1742-4658.2009.07452.x 19909337

[B3] Zhou L Azfer A Niu J Graham S Choudhury M Adamski FM Younce C Binkley PF Kolattukudy PE Monocyte chemoattractant protein-1 induces a novel transcription factor that causes cardiac myocyte apoptosis and ventricular dysfunction Circ Res 2006 98 1177 85 10.1161/01.RES.0000220106.64661.71 16574901 PMC1523425

[B4] Jiang Z Ninomiya-Tsuji J Qian Y Matsumoto K Li X Interleukin-1 (IL-1) receptor-associated kinase-dependent IL-1-induced signaling complexes phosphorylate TAK1 and TAB2 at the plasma membrane and activate TAK1 in the cytosol Mol Cell Biol 2002 22 7158 67 10.1128/MCB.22.20.7158-7167.2002 12242293 PMC139807

[B5] Sharrocks AD The ETS-domain transcription factor family Nat Rev Mol Cell Biol 2001 2 827 37 10.1038/35099076 11715049

[B6] Yang SH Jaffray E Hay RT Sharrocks AD Dynamic interplay of the SUMO and ERK pathways in regulating Elk-1 transcriptional activity Mol Cell 2003 12 63 74 10.1016/S1097-2765(03)00265-X 12887893

[B7] Stevens JL Cantin GT Wang G Shevchenko A Shevchenko A Berk AJ Transcription control by E1A and MAP kinase pathway via Sur2 mediator subunit Science 2002 296 755 8 10.1126/science.1068943 11934987

[B8] Li QJ Yang SH Maeda Y Sladek FM Sharrocks AD Martins-Green M MAP kinase phosphorylation-dependent activation of Elk-1 leads to activation of the co-activator p300 EMBO J 2003 22 281 91 10.1093/emboj/cdg028 12514134 PMC140103

[B9] O'Donnell A Yang SH Sharrocks AD MAP kinase-mediated c-fos regulation relies on a histone acetylation relay switch Mol Cell 2008 29 780 5 10.1016/j.molcel.2008.01.019 18374651 PMC3574235

[B10] Skalniak L Zarebski A Mizgalska D Wyrzykowska P Koj A Jura J NF-kappaB-dependent induction of the MCP-1 induced protein (MCPIP1) transcription upon IL-1 beta stimulation FEBS J 2009 276 5892 905 10.1111/j.1742-4658.2009.07273.x 19747262

[B11] Yang SH Whitmarsh AJ Davis RJ Sharrocks AD Differential targeting of MAP kinases to the ETS-domain transcription factor Elk-1 EMBO J 1998 17 1740 9 10.1093/emboj/17.6.1740 9501095 PMC1170521

[B12] Mountain DJ Singh M Menon B Singh K Interleukin-1beta increases expression and activity of matrix metalloproteinase-2 in cardiac microvascular endothelial cells: role of PKCalpha/beta1 and MAPKs Am J Physiol Cell Physiol 2007 292 C867 75 10.1152/ajpcell.00161.2006 16987994

[B13] Kasza A O'Donnell A Gascoigne K Zeef LA Hayes A Sharrocks AD The ETS domain transcription factor Elk-1 regulates the expression of its partner protein, SRF J Biol Chem 2005 280 1149 55 10.1074/jbc.M411161200 15531578

[B14] Latinkić BV Zeremski M Lau LF Elk-1 can recruit SRF to form a ternary complex upon the serum response element Nucleic Acids Res 1996 24 1345 51 10.1093/nar/24.7.1345 8614640 PMC145793

[B15] Townsend KJ Zhou P Qian L Bieszczad CK Lowrey CH Yen A Craig RW Regulation of MCL1 through a serum response factor/Elk-1-mediated mechanism links expression of a viability-promoting member of the BCL2 family to the induction of hematopoietic cell differentiation J Biol Chem 1999 274 1801 13 10.1074/jbc.274.3.1801 9880563

[B16] Ling Y Lakey JH Roberts CE Sharrocks AD Molecular characterization of the B-box protein-protein interaction motif of the ETS-domain transcription factor Elk-1 EMBO J 1997 16 2431 40 10.1093/emboj/16.9.2431 9171356 PMC1169843

[B17] Boros J Donaldson IJ O'Donnell A Odrowaz ZA Zeef L Lupien M Meyer CA Liu XS Brown M Sharrocks AD Elucidation of the ELK1 target gene network reveals a role in the coordinate regulation of core components of the gene regulation machinery Genome Res 2009 19 1963 1973 10.1101/gr.093047.109 19687146 PMC2775591

[B18] Vickers ER Kasza A Kurnaz IA Seifert A Zeef LA O'donnell A Hayes A Sharrocks AD Ternary complex factor-serum response factor complex-regulated gene activity is required for cellular proliferation and inhibition of apoptotic cell death Mol Cell Biol 2004 24 10340 51 10.1128/MCB.24.23.10340-10351.2004 15542842 PMC529045

[B19] Wurzer WJ Ehrhardt C Pleschka S Berberich-Siebelt F Wolff T Walczak H Planz O Ludwig S NF-kappaB-dependent induction of tumor necrosis factor-related apoptosis-inducing ligand (TRAIL) and Fas/FasL is crucial for efficient influenza virus propagation J Biol Chem 2004 279 30931 30937 10.1074/jbc.M403258200 15143063

[B20] Kubica M Guzik K Koziel J Zarebski M Richter W Gajkowska B Golda A Maciag-Gudowska A Brix K Shaw L Foster T Potempa J A potential new pathway for *Staphylococcus aureus *dissemination: the silent survival of *S. aureus *phagocytosed by human monocyte-derived macrophages PLoS ONE 2008 3 e1409 10.1371/journal.pone.0001409 18183290 PMC2169301

[B21] Jura J Wegrzyn P Zarebski A Władyka B Koj A Identification of changes in the transcriptome profile of human hepatoma HepG2 cells stimulated with interleukin-1 beta Biochim Biophys Acta 2004 1689 120 33 15196593 10.1016/j.bbadis.2004.03.002

[B22] Kasza A Kiss DL Goplan S Xu W Rydel RE Koj A Kordula T Mechanism of plasminogen activator inhibitor-1 regulation by oncostatin M and interleukin-1 in human astrocytes J Neurochem 2002 83 696 703 10.1046/j.1471-4159.2002.01163.x 12390531 PMC4567031

