# Supplementary material for: Transcription factors Elk-1 and SRF are engaged in IL1-dependent regulation of ZC3H12A expression
Source: BMC Mol Biol. 2010 Feb 6;11:14. doi: 10.1186/1471-2199-11-14 (PMC2829564; doi:10.1186/1471-2199-11-14)
Supplement: Additional file 1 — Fig. S1. Activation of NFκB in HepG2, MOCK and mIκB cells. Nuclear extract was isolated from HepG2 cells, MOCK cells and mIκB cells. Where indicated cells were stimulated with IL-1β (15 ng/ml). By gel retardation assay NFκB activation was measured. [file 1471-2199-11-14-S1.PPT]

## Slide 1
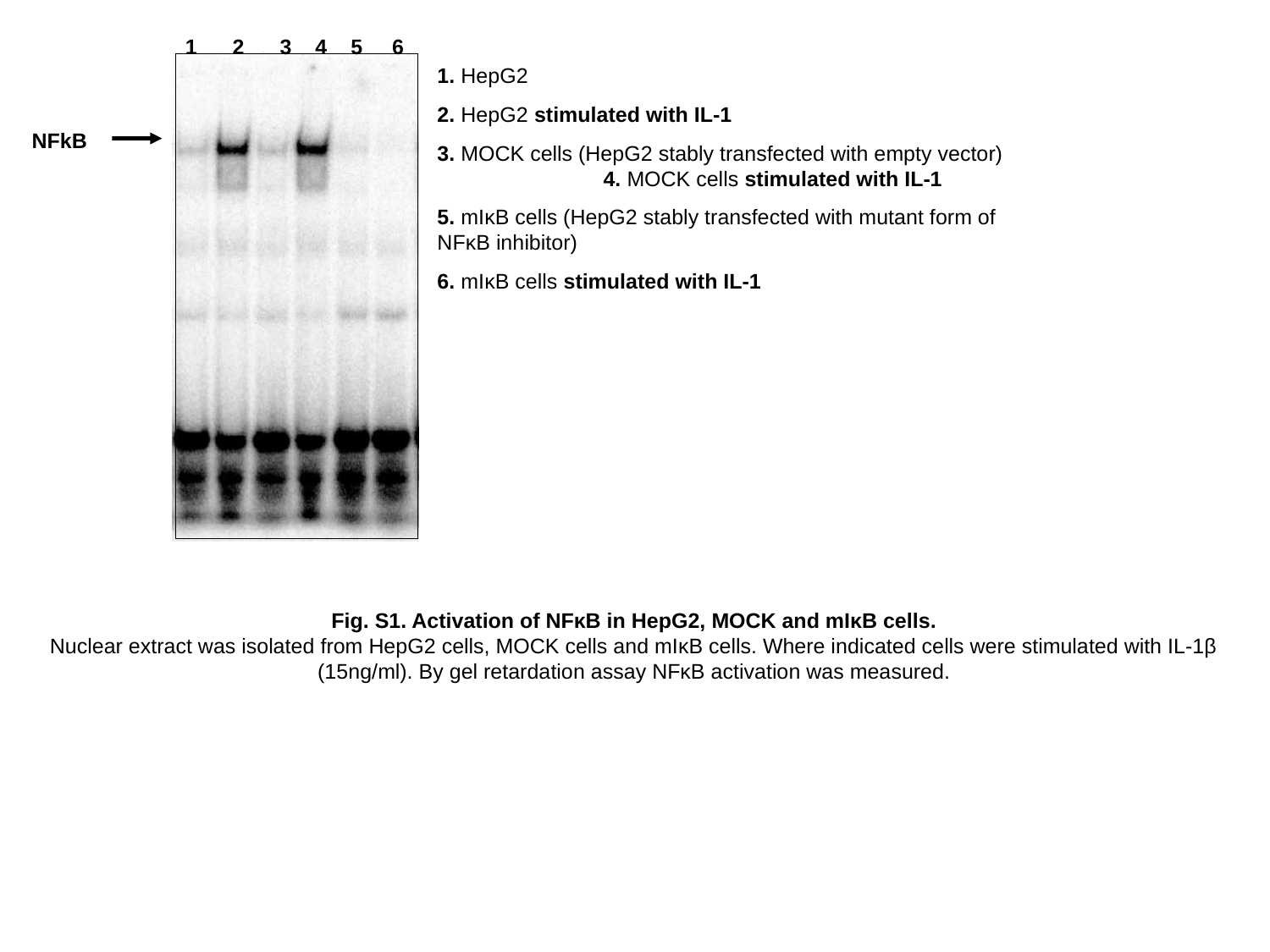

1 2 3 4 5 6
1. HepG2
2. HepG2 stimulated with IL-1
3. MOCK cells (HepG2 stably transfected with empty vector) 4. MOCK cells stimulated with IL-1
5. mIκB cells (HepG2 stably transfected with mutant form of NFκB inhibitor)
6. mIκB cells stimulated with IL-1
NFkB
Fig. S1. Activation of NFκB in HepG2, MOCK and mIκB cells.
Nuclear extract was isolated from HepG2 cells, MOCK cells and mIκB cells. Where indicated cells were stimulated with IL-1β (15ng/ml). By gel retardation assay NFκB activation was measured.
