# Supplementary material for: Transcription factors Elk-1 and SRF are engaged in IL1-dependent regulation of ZC3H12A expression
Source: BMC Mol Biol. 2010 Feb 6;11:14. doi: 10.1186/1471-2199-11-14 (PMC2829564; doi:10.1186/1471-2199-11-14)
Supplement: Additional file 2 — Fig. S2. Activation of ERK by IL-1β in HepG2 cells. HepG2 cells were stimulated by IL-1β (15 ng/ml) for 15 and 30 min and the phosphorylation of ERK1/2 was analyzed by western blot analysis. To some experimental groups U0126 (10 μM) was added 30 min prior IL-1β stimulation (lanes 4-6) [file 1471-2199-11-14-S2.PPT]

## Slide 1
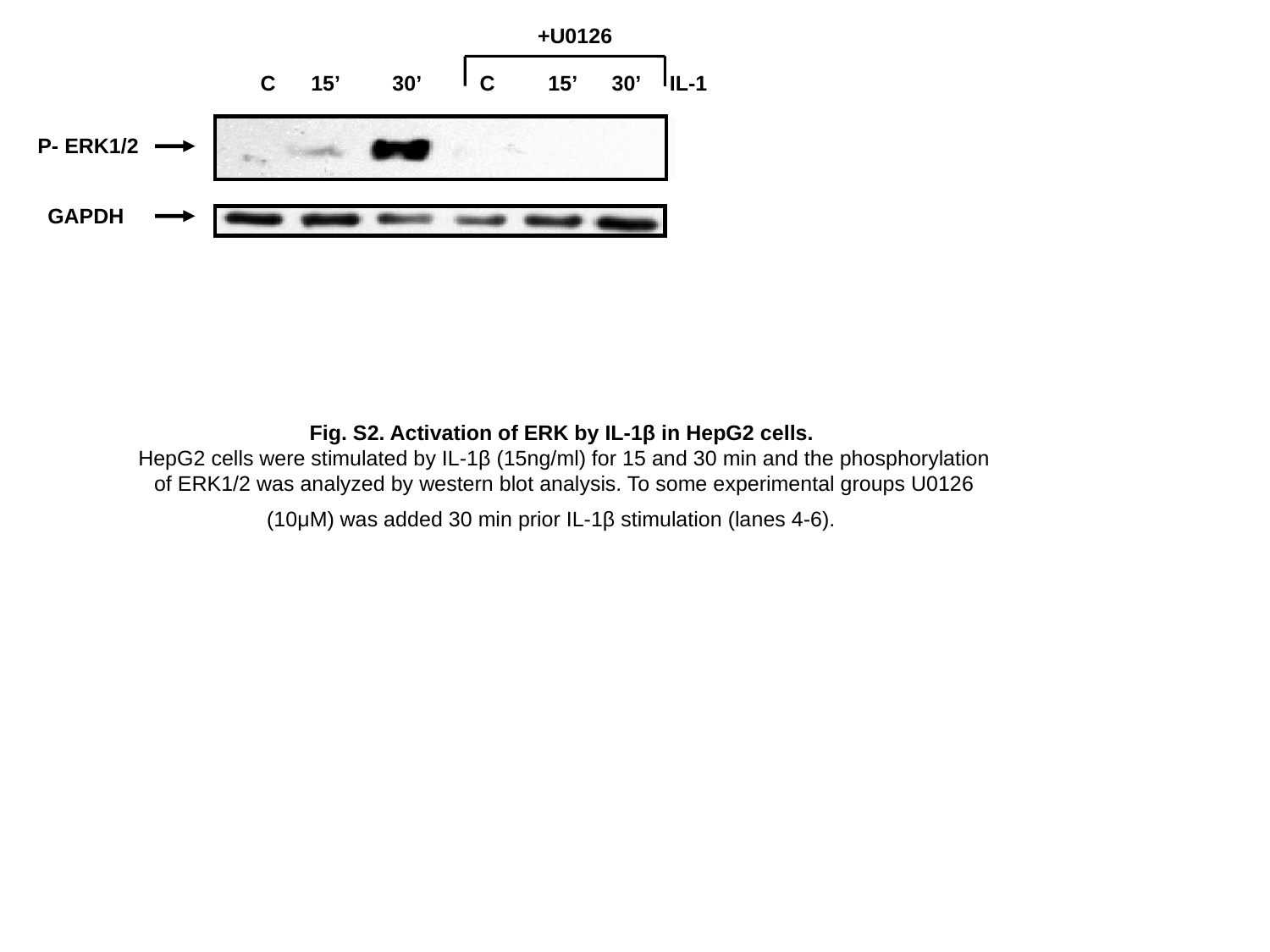

+U0126
C 15’ 30’ C 15’ 30’ IL-1
P- ERK1/2
GAPDH
Fig. S2. Activation of ERK by IL-1β in HepG2 cells.
HepG2 cells were stimulated by IL-1β (15ng/ml) for 15 and 30 min and the phosphorylation of ERK1/2 was analyzed by western blot analysis. To some experimental groups U0126 (10μM) was added 30 min prior IL-1β stimulation (lanes 4-6).
