# Supplementary material for: Transcription factors Elk-1 and SRF are engaged in IL1-dependent regulation of ZC3H12A expression
Source: BMC Mol Biol. 2010 Feb 6;11:14. doi: 10.1186/1471-2199-11-14 (PMC2829564; doi:10.1186/1471-2199-11-14)
Supplement: Additional file 3 — Fig. S3. Regulation of 2038 bp length ZC3H12A promoter fragment by Elk-1. HepG2 cells were transiently transfected with the luciferase construct containing fragment of human ZC3H12A promoter located between -1050 and +988 and increasing amounts of pElk-En (20, 50, 100, 200 ng - lanes 2, 3, 4, 5) or Elk-VP16 (20, 50, 100, 200 ng - lanes 6, 7, 8, 9). Lane 1 - control cells without Elk-En or Elk-VP16. Luciferase activity was measured 24 h after transfection. Statistical significance was determined using the Student's test. *P < 0.05, **P < 0.001. [file 1471-2199-11-14-S3.PPT]

## Slide 1
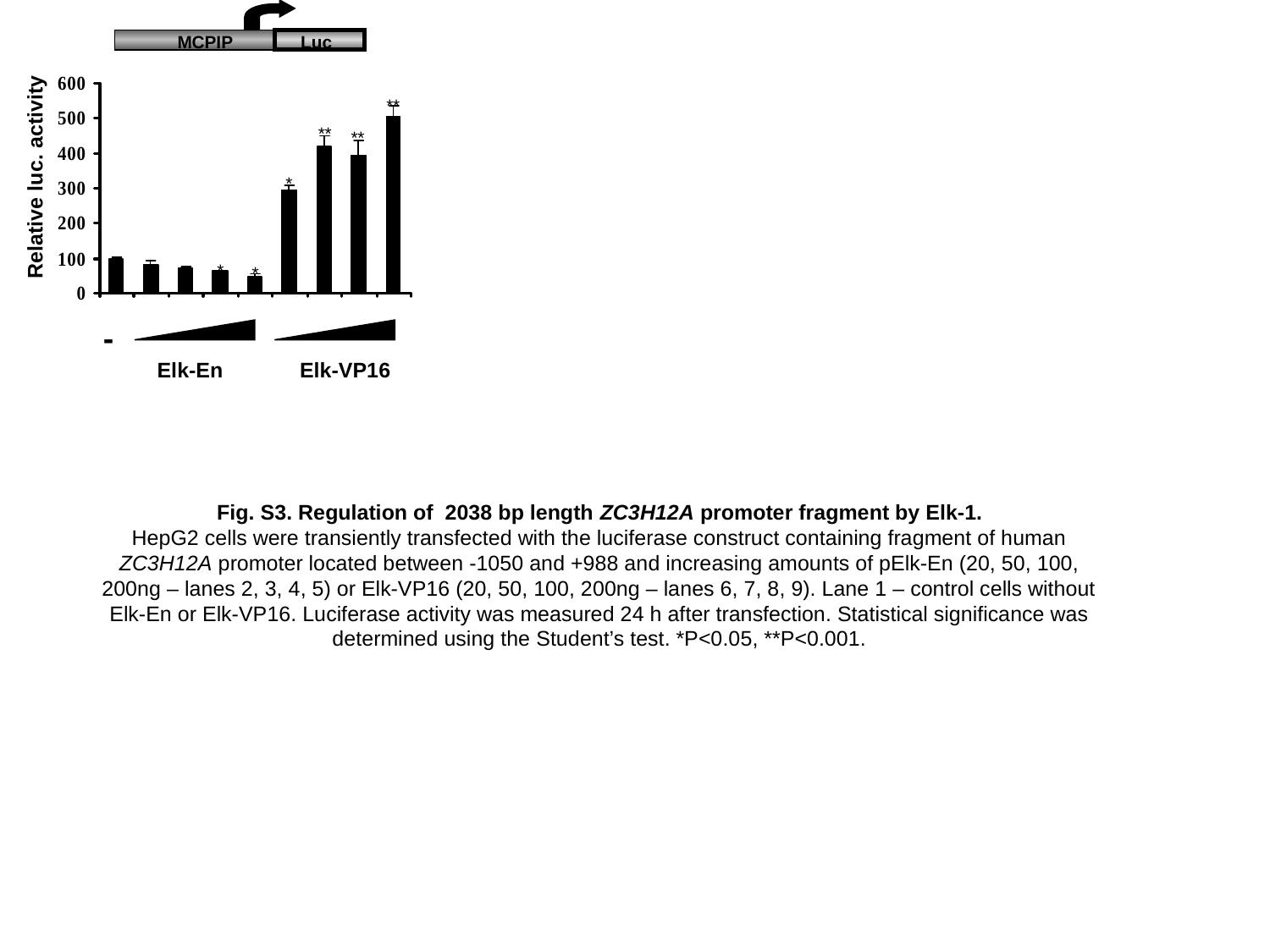

MCPIP Luc
Relative luc. activity
-
Elk-En Elk-VP16
**
**
**
*
*
*
Fig. S3. Regulation of 2038 bp length ZC3H12A promoter fragment by Elk-1.
HepG2 cells were transiently transfected with the luciferase construct containing fragment of human ZC3H12A promoter located between -1050 and +988 and increasing amounts of pElk-En (20, 50, 100, 200ng – lanes 2, 3, 4, 5) or Elk-VP16 (20, 50, 100, 200ng – lanes 6, 7, 8, 9). Lane 1 – control cells without Elk-En or Elk-VP16. Luciferase activity was measured 24 h after transfection. Statistical significance was determined using the Student’s test. *P<0.05, **P<0.001.
